# Diabetes-related distress and psychological burden in patients with type 1 and type 2 diabetes – the role of child maltreatment, personality functioning, and epistemic trust: findings from a German clinical inpatient sample

**DOI:** 10.3389/fpsyt.2025.1608601

**Published:** 2025-07-17

**Authors:** Sandra Zara, Imogen Carla Ullrich, Anna Maria Leveling, Friederike Gisela Genoveva Saretzki, Jana Schmitz, Michael Eckhard, Johannes Kruse, Hanna Kampling

**Affiliations:** ^1^ Department of Psychosomatic Medicine and Psychotherapy, Justus Liebig University Gissen, Giessen, Germany; ^2^ GZW Diabetes-Klinik Bad Nauheim, Bad Nauheim, Germany; ^3^ Department for Psychosomatic Medicine and Psychotherapy, Medical Center of the Philipps University, Marburg, Germany; ^4^ German Center for Diabetes Research (DZD), Neuherberg, Germany

**Keywords:** child maltreatment, clinical sample, diabetes-related distress, epistemic trust, personality functioning, psychological burden

## Abstract

**Introduction:**

Patients with diabetes exhibit high frequencies of psychological burden and diabetes-related distress. Child maltreatment has been independently linked to both diabetes and psychological burden. We aimed 1) to explore the association between child maltreatment and diabetes-related distress, and between child maltreatment and psychological burden, and 2) to investigate the mediating role of personality functioning (psychological abilities with regard to the self and others) in this association. We included epistemic trust (openness for social learning) and its impairments mistrust (the tendency to consider information sources as unreliable or ill-intended) and credulity (lack of vigilance and discrimination between trustworthy and untrustworthy information) as covariates.

**Methods:**

In a sample of n=119 patients with type 1 or 2 diabetes aged 18-69, self-report questionnaires assessed diabetes-related distress (PAID-5), psychological burden (PHQ-D, PHQ-9, PHQ-15), child maltreatment (CTQ), personality functioning (OPD-SQS), and epistemic trust, mistrust, and credulity (ETMCQ). Mediation analyses assessed the role of personality functioning in the association between child maltreatment, diabetes-related distress, and psychological burden, including epistemic trust, mistrust, and credulity as covariates.

**Results:**

Patients with child maltreatment compared to those without showed higher diabetes-related distress (*t*_(112)_=4.033, *p*<.001, *d*=.756) and higher frequencies of major depressive syndrome (χ^2^_(1)_=10.968, *p*<.001, φ=.310) with medium effect sizes. They showed higher frequencies of somatoform syndrome (χ^2^_(1)_=8.130, *p*=.004, φ=.267), panic syndrome (χ^2^_(1)_=6.281, *p*=.012, φ=.235), and other anxiety syndromes (χ^2^_(1)_=8.828, *p*=.003, φ=.278), with small effect sizes. Impairments in personality functioning were more severe (*t*_(100,857)_=5.136, *p*<.001, d=.967), with a large effect size. Epistemic mistrust and credulity were significantly higher (mistrust: *t*_(111)_=3.677, *p*<.001, d=.692; credulity: *t*_(111)_=5.000, *p*<.001, d=.941), with medium and large effect sizes. No differences regarding epistemic trust were found (*t*_(111)_=.409, *p=*.683, d=.077), with the effect size below the threshold for small effects. Personality functioning mediated the association between child maltreatment and diabetes-related distress (b=.03, 95%-CI [.005,.053]), depressive symptoms (b=.08, 95%-CI [.030,.129]), and somatic symptoms (b=.06, 95%-CI [.020,.122]).

**Conclusions:**

In a clinical diabetes sample, diabetes-related distress and psychological burden are frequently reported, and personality functioning is impaired, particularly after child maltreatment. Addressing these impairments and initiating psychosomatic treatment including psychodynamic or mentalization-based interventions might offer new clinical treatment avenues.

## 1 Introduction

Diabetes mellitus is a chronic non-communicable disease characterized by steadily heightened blood glucose levels, affecting up to 11% of the German population (1). It is associated with increased morbidity, mortality, and impaired quality of life, which is primarily attributable to somatic secondary diseases as well as high psychological burden (2, 3). The most common diabetes types are type 1 and type 2 diabetes. While type 1 diabetes is characterized by an autoimmune destruction of the insulin-producing β-cells in the pancreas, potentially resulting in a severe underproduction or even a complete lack of insulin (4, 5), type 2 diabetes is linked to lifestyle factors such as obesity, unfavourable diet, and lack of exercise. Type 2 diabetes accounts for around 90% of diabetes cases (6).

Diabetes-related distress and psychological burden in terms of e.g., depressive and anxiety symptoms or phenomena linked to psychological burden, such as suicidal ideation and loneliness, are highly prevalent in patients with diabetes (7–10). Studies show that diabetes-related distress and psychological burden are independently from one another associated with impaired diabetes self-management, diabetes-related complications, increased morbidity and mortality, reduced quality of life as well as increased health care costs (11–15). Further, individuals with diabetes compared to the general population are at nearly twice the risk of developing depressive and anxiety symptoms (16, 17), and approximately 22% of patients with type 1 and 36% of patients with type 2 diabetes report elevated diabetes-related distress (18, 19).

One psychosocial aspect gaining more prominence in diabetes research is adverse childhood experiences (ACEs). ACEs comprise sexual, physical and emotional abuse, physical and emotional neglect as well as household dysfunctions (e.g., loss of a parent, alcohol/drug abuse) (20, 21). While around 20% of the German general population report at least one type of ACE of at least moderate severity, among individuals with diabetes the frequency of ACEs is considerably higher with up to 32% (22). ACEs appear to be a risk factor for the onset of diabetes (23–25), and among individuals with diabetes they are also associated with higher depression and anxiety symptoms, stronger impairments in diabetes self-management, a less frequent utilization of diabetes care, higher blood sugar levels, and more frequent suicidal ideation (9, 26, 27).

With the intertwined negative implications for patients with diabetes regarding ACEs, diabetes-related distress, and psychological burden, it is necessary to better understand their underlying and shared mechanisms. While little is known regarding diabetes-related distress, other lines of research increasingly employ the transdiagnostic constructs of personality functioning and the epistemic stance comprising epistemic trust, mistrust, and credulity and its relevance in understanding the association between ACEs and psychological burden (28, 29). Both personality functioning and the epistemic stance develop in early childhood in the context of secure attachment experiences (30) and can be impaired by ACEs. Personality functioning (also called structure) as defined by the Operationalized Psychodynamic Diagnosis System (OPD), describes a person’s basic psychological abilities with regard to the self, e.g., identity and self-esteem, and in relation to others, e.g., empathy and intimacy in the four domains cognition/perception, regulation, communication, and attachment (31). This allows for a differentiated and dimensional assessment of a person’s psychological abilities or personality functioning respectively. Accordingly, related – but distinct – approaches to dimensionally assess primarily personality disorders have been considered in the Alternative Model of Personality Disorders (AMPD) in the 5^th^ edition of the Diagnostic and Statistical Manual of Mental Disorders (DSM-5) (32) as well as the ICD-11, replacing a solely categorical assessment of personality disorders by a dimensional severity rating (33, 34). Impairments in personality functioning were linked to numerous psychological burden e.g., depression and anxiety symptoms, (complex) posttraumatic stress disorder symptoms, and somatic symptom disorder (28, 29, 35). Closely related to personality functioning is the epistemic stance, with epistemic trust describing a persons’ openness for social learning opportunities and trust in socially transmitted information (36). Disruptions of epistemic trust might result in epistemic mistrust, which is the tendency to judge sources of information as not credible and avoiding influence by socially mediated information, or epistemic credulity, describing the susceptibility to misinformation and vulnerability for exploitation by others (37). Epistemic trust is crucial for a healthy development of personality functioning and might be highly relevant for the patient-physician-relationship, as trusting information, especially of diabetologists, is a key element in successful diabetes treatment. Diabetes research has only recently picked up on the importance of personality functioning and demonstrated based on a representative population sample that impairments in personality functioning mediate the association between child abuse and diabetes (22). Further, impaired personality functioning rather than depressive symptoms are associated with a less steep decline of blood sugar levels during a six month standardized disease management program (38), indicating the relevance of personality functioning to better understand associated detrimental effects of ACEs and psychological burden in patients with diabetes.

Against this background, the aim of this study is a) to add evidence to the associations between ACEs and diabetes-related distress, and ACEs and psychological burden, and b) expand the knowledge on personality functioning and epistemic trust, mistrust, and credulity in a clinical sample of patients with type 1 and type 2 diabetes. Employing the construct of child maltreatment comprising sexual, physical, emotional abuse as well as physical and emotional neglect, we investigated the following research questions:

- Do patients with diabetes and a history of child maltreatment compared to those without child maltreatment show higher diabetes-related distress and more frequent psychological burden?
- Do patients with diabetes and a history of child maltreatment compared to those without child maltreatment show higher impairments in personality functioning and epistemic trust, mistrust, and credulity?
- Is the association between child maltreatment and diabetes-related distress, as well as between child maltreatment and psychological burden, mediated by personality functioning, including epistemic trust, mistrust, and credulity as covariates?

## 2 Methods

### 2.1 Sample and setting

Patients with medically diagnosed diabetes mellitus type 1 or type 2 were recruited between August 2022 and October 2024 in a specialized diabetes clinic in Germany. Demographics and clinical data were obtained from routine medical records of the hospital at the point of admission. Inclusion criteria comprised an age between 18 and 69, blood sugar levels (HbA_1c_) > 58 mmol/mol (7.5%), at least 2 years since the diabetes diagnosis, having participated in a diabetes training, and were therefore expected to be sufficiently educated and capable to deal with their diabetes. Further, sufficient German language skills and cognitive ability were required. Exclusion criteria comprised patients with a type 3 diabetes or gestation diabetes, severe comorbid illnesses, e.g., severe depressive episodes, or dementia. This investigation is part of the project “Ed-iPP: Capturing Diabetes-Related Distress and Burden From the Perspective of Patients With Type 1 or Type 2 Diabetes”, which comprised qualitative assessments in phase 1) and quantitative assessments in phase 2). In phase 1) n=40 patients with type 1 and type 2 diabetes and in phase 2) n=100 patients with type 1 and type 2 diabetes were included in the study, resulting in a total of n=140. However, due to not meeting inclusion criteria or retracting consent, n=21 patients were excluded from the study, resulting in a final analyses sample of n=119 patients. The final analyses sample comprised n=57 (47.9%) patients with type 1 and n=62 (52.1%) patients with type 2 diabetes. In the total sample, the mean age was 49.4 years. With 65.0%, the percentage of males was higher than the percentage of females. The present study focuses on quantitative analyses. The study protocol can be found elsewhere (39). The Ed-iPP-study was approved by the ethics committee of the Justus Liebig University Giessen (AZ 161/21) and was conducted according to the applicable COVID-19 restrictions as well as the German General Data Protection Regulation (DSGVO) and the State and Federal Data Protection Act (LDSG and BDSG).

### 2.2 Assessment

#### 2.2.1 Child maltreatment – childhood trauma questionnaire

The CTQ retrospectively assesses sexual, emotional and physical abuse as well as emotional and physical neglect with 5 items per subscale. Answers range from 1=‘not at all’ to 5=‘very often’, with sum scores ranging from 5 to 25 for each subscale and 25 to 125 for the total scale. The severity of each type of child maltreatment was calculated using norm data, resulting in four severity levels: ‘none to minimal’, ‘minimal to moderate’, ‘moderate to severe’ and ‘severe to extreme’ (40). Dichotomous categories of child maltreatment were created, with scores reflecting at least moderate severity indicating the presence of the respective type of child maltreatment (≥ 8 for sexual abuse, ≥ 10 for physical abuse, ≥ 13 for emotional abuse, ≥ 10 for physical neglect, and ≥ 15 for emotional neglect) (41, 42). These cut-off scores to indicate the presence of child maltreatment are widely used in German prevalence studies (43, 44). The 5-factor structure has been confirmed for the German version, showing good validity and internal consistency for all subscales (α=.80-.89), except for physical neglect (α=.55) (40). In the present sample, internal consistency for the total scale was excellent (α=.92), and ranged between (α=.60-.95) for the subscales.

#### 2.2.2 Diabetes-related distress – problem areas in diabetes scale (PAID-5)

The PAID-5 addresses burden regarding diabetes self-management consisting of 5 Items (45), with response options ranging from 0=‘no problem’ to 4=‘big problem’. The sum score ranges between 0 and 20, a cut-off-value of ≥ 11 indicates high diabetes-related distress (46). The PAID-5 shows satisfactory sensitivity (94%) and specificity (89%). The German version has shown good internal consistency α=.83 (46). In the present sample, internal consistency for the total scale was good (α=.86).

#### 2.2.3 Psychological burden – patient-health-questionnaire

The PHQ-D assesses numerous psychopathologies according to DSM-IV criteria and has proven to be a valid and reliable screening measure for major depressive syndrome, somatoform syndrome, panic syndrome, other anxiety syndromes, bulimia nervosa, binge-eating disorder, and alcohol syndrome (47, 48). Each psychopathology can be categorically evaluated. Moreover, depressive symptoms as well as somatic symptoms can be dimensionally assessed with the PHQ-9 and PHQ-15 respectively as part of the PHQ-D. The PHQ-9 comprises 9 items addressing depressive symptoms, with response options ranging from 0=‘not at all’ to 3=‘nearly every day’ (49). The sum score ranges from 0 to 27, with values from 5–9 indicating mild, values from 10–14 indicating moderate, and values from 15–27 indicating severe depressive symptoms (49). The PHQ-15 contains 15 items addressing somatic complaints with response options ranging from 0=‘not bothered at all’ to 2=‘bothered a lot’, as well as two items of the PHQ-9 addressing sleep quality and energy levels. The total score ranges from 0 to 30 (50). The severity of somatic symptoms can be assessed as minimal (0–4), low (5-9), medium (10-14), or high (15-30) (50). The PHQ-D has shown good validity, especially for major depressive syndrome (48). In the present sample, the internal consistency for each PHQ-D scale ranged from α=.94-.81 (PHQ-9: α=.91; PHQ-15: α=.82), with the weakest internal consistency being observed for bulimia nervosa (α=.55) and alcohol syndrome (α=.43).

#### 2.2.4 Personality functioning – operationalized psychodynamic diagnosis–structure questionnaire short form

Personality functioning and related impairments as defined by the Operationalized Psychodynamic Diagnosis System are assessed with the OPD-SQS (31, 51, 52). It comprises three subscales (self-perception, interpersonal contact, and relationship model) with four items each. Response options range from 0=‘does not apply at all’ to 4=‘fully applies’, resulting in a sum score ranging from 0 to 48. Higher scores indicate more severe deficits in personality functioning. The OPD-SQS showed good factor validity, convergent validity, and internal consistency (α=.88) (52). In the present study, the internal consistency of the total scale is α=.91.

#### 2.2.5 Epistemic trust, mistrust, and credulity – epistemic trust, mistrust and credulity questionnaire

The epistemic stance comprising epistemic trust, mistrust, and credulity is assessed with the German 12-item self-report questionnaire ETMCQ (53). Response options range from 1=‘strongly disagree’ to 7=‘strongly agree’, with higher scores indicating a stronger endorsement of trust, mistrust, or credulity. The subscales comprise five items for trust, three items for mistrust, and four items for credulity, with sum scores ranging from 5 to 35, 3 to 21, and 4 to 28 respectively (53). Good to acceptable internal consistency has been proposed for the German version (trust: α=.81; ω=.81; mistrust: α=.65; ω=.66; credulity: α=.81; ω=.81) (53). In the present sample, the subscales showed acceptable internal consistency with ω=.69 for epistemic trust, ω=.76 for mistrust, and ω=.79 for credulity.

### 2.3 Statistical analyses

In order to investigate group differences between patients with diabetes with and without child maltreatment, a dichotomous child maltreatment variable was calculated. If a patient reported at least one type of child maltreatment of at least moderate severity, child maltreatment was evaluated as present. No child maltreatment was defined as not reporting any type of child maltreatment. Differences were investigated regarding diabetes-related distress, psychological burden, personality functioning, and epistemic trust, mistrust, and credulity, and examined with *t*-tests and Chi^2^-tests. Regarding effect sizes, Phi (φ) was reported for 2x2 contingency tables, Cramer’s V for non 2x2 contingency tables, and Cohen’s d for independent *t*-Tests. Phi and Cramer’s V values of.1,.3, and.5 are considered small, medium, and large effects respectively (54). Cohen’s d of.2,.5, and.8 is considered a small, medium, and large effect respectively (54). Mediation analyses tested the associations between child maltreatment, personality functioning, and diabetes-related distress as well as between child maltreatment, personality functioning, and psychological burden in terms of depressive and somatic symptoms (PHQ-9 and PHQ-15) including epistemic trust, mistrust, and credulity as covariates. We followed two steps: first, the direct association between child maltreatment and diabetes-related distress and between child maltreatment and psychological burden respectively, was examined. Next, a mediation model employing personality functioning as a mediator and epistemic trust, mistrust, and credulity as covariates was tested. Model fit was examined with adjusted R^2^, interpreted as small (R^2^=.02), medium (R^2^=.13), or large (R^2^=.26) (54). The significance of the associations was examined using bootstrapped confidence intervals (5,000 samples, 95%-confidence intervals (CI)). Mediation analyses were conducted with the PROCESS macro (v4.2) for IBM SPSS Statistics (55). Child maltreatment (CTQ sum score) was added as independent variable, personality functioning (OPD-SQS sum score) was added as a mediator, and diabetes-related distress (PAID-5 sum score) and psychological burden (depressive symptoms: PHQ-9 sum score; somatic symptoms: PHQ-15 sum score) were added as dependent variable respectively. Epistemic trust, mistrust, and credulity (ETMCQ-12 subscale sum scores) were added as covariates. A mediating effect is present, if the CIs of the regression weight exclude zero. Significance levels were set at *p* <.05. All analyses were conducted in IBM SPSS Statistics v29.

## 3 Results

### 3.1 Sociodemographic and clinical characteristics

In the total sample, 32.8% (type 1: 29.8%, type 2: 35.5%) reported elevated diabetes-related distress (PAID-5 ≥ 11). Moreover, 53.8% (type 1: 52.6%, type 2: 54.8%) met the categorical self-report threshold for at least one psychopathology based on the PHQ-D, and 26.9% met the threshold for two or more types of psychopathology. The most frequently reported psychological burden was major depressive syndrome (32.8%), followed by somatoform syndrome (26.9%), anxiety syndromes (15.1%), binge-eating disorder (12.6%), panic syndrome (10.9%), alcohol syndrome (10.1%), and bulimia nervosa (2.5%). Regarding the severity of depressive and somatic symptoms (PHQ-9 and PHQ-15), 10.0% of patients reported severe depressive symptoms and 28.3% reported severe somatic symptoms. Regarding child maltreatment, 49.1% self-reported at least one type of at least moderate abuse or neglect, and 37.0% reported at least one type of severe abuse or neglect (CTQ). The most frequently self-reported type of child maltreatment was physical neglect (31.0%), closely followed by emotional neglect (30.2%), emotional abuse (23.9%), physical abuse (16.7%), and sexual abuse (8.7%).

Comparing the diabetes types showed that patients with type 2 diabetes were significantly older compared to patients with type 1 diabetes (*t* (_84,174_)=8.573, *p* <.001, d=1.604), with a large effect size. The percentage of male participants was higher in patients with type 2 diabetes compared to type 1 diabetes (χ^2^_(1)_=6.984, *p=*.008, φ=.242), with a small effect size. Regarding diabetes-related distress, the frequency of any type of self-reported psychological burden, as well as the severity of depressive and somatic symptoms there were no differences. Regarding different types of child maltreatment, patients with type 2 diabetes reported significantly more often physical abuse (χ^2^_(1)_=4.391, *p=*.036, φ=.196), and emotional neglect (χ^2^_(1)_=3.929, *p=*.047, φ=.184), with small effect sizes. Regarding personality functioning and epistemic trust, no differences emerged. For more details, see **Table 1**.

**Table 1:** Sociodemographic and clinical characteristics.

	Total sample (n = 119)		Type 1 diabetes^a^ (n = 57)		Type 2 diabetes^a^ (n = 62)		Test statistics	Effect size
	N	(%)	N	(%)	N	(%)		
Gender								
male	77	(65.0)	30	(52.6)	47	(75.8)	χ^2^_(1)_ = 6.984, *p* = .008	φ = .242
female	42	(35.0)	27	(47.4)	15	(24.2)		
Psychological burden^b^								
major depressive syndrome	39	(32.8)	17	(29.8)	22	(35.5)	χ^2^_(1)_ = .432, *p* = .511	φ = .060
somatoform syndrome	32	(26.9)	12	(21.1)	20	(32.3)	χ^2^_(1)_ = 1.897, *p* = .168	φ = .126
panic syndrome	13	(10.9)	5	(8.8)	8	(12.9)	χ^2^_(1)_ = .521, *p* = .470	φ = .066
other anxiety syndromes	18	(15.1)	5	(8.8)	13	(21.0)	χ^2^_(1)_ = 3.441, *p* = .064	φ = .170
bulimia nervosa	3	(2.5)	0	(0.0)	3	(4.8)	n.a.^c^	
binge-eating disorder	15	(12.6)	8	(14.3)	7	(13.0)	χ^2^_(1)_ = .041, *p* = .840	φ = .019
alcohol syndrome	12	(10.1)	8	(14.0)	4	(6.5)	χ^2^_(1)_ = 1.884, *p* = .170	φ = .126
Depressive symptom severity^d^								
minimal	36	(32.7)	19	(34.5)	17	(30.9)	χ^2^_(4)_ = 1.579, *p* = .812	Cramer’s V = .120
mild	29	(26.4)	15	(27.3)	14	(25.5)		
moderate	20	(18.2)	11	(20.0)	9	(16.4)		
moderately severe	14	(12.7)	5	(9.1)	9	(16.4)		
severe	11	(10.9)	5	(9.1)	6	(10.9)		
Somatic symptom severity^e^								
minimal	19	(20.7)	13	(26.0)	6	(14.3)	χ^2^_(3)_ = 2.182, *p* = .535	Cramer’s V = .154
mild	31	(33.7)	15	(30.0)	16	(38.1)		
moderate	16	(17.4)	9	(18.0)	7	(16.7)		
severe	26	(28.3)	13	(26.0)	13	(31.0)		
Child maltreatment^f^								
any type	56	(49.1)	24	(43.6)	32	(54.2)	χ^2^_(1)_ = 1.280, *p* = .258	φ = .106
sexual abuse	10	(8.7)	5	(8.9)	5	(8.5)	n.a.^c^	
physical abuse	19	(16.7)	5	(9.1)	14	(23.7)	χ^2^_(1)_ = 4.391, *p* = .036	φ = .196
emotional abuse	27	(23.9)	12	(21.8)	15	(25.9)	χ^2^_(1)_ = .254, *p* = .614	φ = .047
physical neglect	36	(31.0)	13	(23.2)	23	(38.3)	χ^2^_(1)_ = 3.094, *p* = .079	φ = .163
emotional neglect	35	(30.2)	12	(21.4)	23	(38.3)	χ^2^_(1)_ = 3.929, *p* = .047	φ = .184
	M	(SD)	M	(SD)	M	(SD)		
age	49.4	(13.0)	40.9	(12.8)	57.2	(6.9)	*t*_(84,174)_ = 8.573, *p* <.001	d = 1.604
diabetes-related distress^g^	8.6	(5.3)	8.3	(4.9)	9.0	(5.7)	*t*_(117)_ = .650, *p* = .517	d = .119
personality functioning^h^	18.6	(11.3)	18.7	(10.7)	18.5	(11.9)	*t*_(118)_ = -.092, *p* = .927	d = .018
epistemic trust^i^	24.4	(5.4)	24.8	(5.1)	24.1	(5.7)	*t*_(116)_ = -.725, *p* = .470	d = .111
epistemic mistrust^i^	12.0	(4.4)	11.6	(3.9)	12.5	(4.9)	*t*_(111,490)_ = 1.148, *p* = .254	d = .211
epistemic credulity^i^	13.5	(5.7)	12.9	(5.0)	14.0	(6.2)	*t*_(116)_ = 1.077, *p* = .284	d = .192

a Medically diagnosed type 1 or type 2 diabetes. ^b^Psychological burden was assessed with the German version of the Patient-Health-Questionnaire (PHQ-D). ^c^Given that the expected cell frequencies were less than five, the χ^2^-test could not be interpreted. ^d^Depressive symptoms and their severity level were assessed with the PHQ-9 as part of the PHQ-D. Range: 0 to 27. ^e^Somatic symptoms and their severity level were assessed with the PHQ-15 as part of the PHQ-D. Range: 0 to 30. ^f^Different types of child maltreatment were assessed with the self-report questionnaire Childhood Trauma Questionnaire (CTQ). Multiple answers were possible. ^g^Diabetes-related distress was assessed with the 5-item short form of the Problem Areas in Diabetes Scale (PAID-5). Range: 0 to 20. ^h^Personality functioning was assessed with the Operationalized Psychodynamic Diagnosis – Structure Questionnaire Short Form (OPD-SQS). Range: 0 to 48. ^i^Epistemic trust, epistemic mistrust, and epistemic credulity were assessed with the German 12-item version of the Epistemic Trust, Mistrust and Credulity Questionnaire (ETMCQ). Range trust: 5 to 35, range mistrust: 3 to 21, range credulity: 4 to 28. n.a.= not available.

### 3.2 Differences in diabetes-related distress and psychological burden according to a history of child maltreatment

In the total sample, we observed overall higher diabetes-related distress in patients with child maltreatment compared to those without child maltreatment with medium effect sizes (*t*_(112)_=4.033, *p <*.001, d=.756) as well as higher frequencies of psychological burden (major depressive syndrome: χ^2^_(1)_=10.968, *p* <.001, φ=.310, somatoform syndrome: χ^2^_(1)_=8.130, *p*=.004, φ=.267, panic syndrome: χ^2^_(1)_=6.281, *p*=.012, φ=.235, and other anxiety syndromes: χ^2^_(1)_=8.828, *p*=.003, φ=.278), with a medium effect size for major depressive syndrome and small effect sizes for all other types of psychological burden. There were no differences regarding binge-eating disorder (χ^2^_(1)_=.699, *p*=.403, φ=.081) and alcohol syndrome (χ^2^_(1)_=.004, *p*=.949, φ=.006), with effect sizes falling below the threshold for small effects. See also **Table 2**.

**Table 2:** Differences regarding diabetes-related distress, psychological burden as well as personality functioning and epistemic trust, mistrust, and credulity according to a history of child maltreatment.

Psychological burden^b^	Total sample (n = 114)		Child maltreatment^a^ (n = 56)		No child maltreatment (n = 58)		Test statistics	Effect size
	N	(%)	N	(%)	N	(%)		
major depressive syndrome	38	(33.3)	27	(48.2)	11	(19.0)	χ^2^_(1)_ = 10.968, *p* <.001	φ = .310
somatoform syndrome	31	(27.2)	22	(39.3)	9	(15.5)	χ^2^_(1)_ = 8.130, *p* = .004	φ = .267
panic syndrome	12	(10.5)	10	(17.9)	2	(3.4)	χ^2^_(1)_ = 6.281, *p* = .012	φ = .235
other anxiety syndromes	17	(14.9)	14	(25.0)	3	(5.2)	χ^2^_(1)_ = 8.828, *p* = .003	φ = .278
bulimia nervosa	2	(1.8)	0	(0.0)	2	(3.6)	n.a.^c^	
binge-eating disorder	15	(14.2)	9	(17.0)	6	(11.3)	χ^2^_(1)_ = .699, *p* = .403	φ = .081
alcohol syndrome	12	(10.)	6	(10.7)	6	(10.3)	χ^2^_(1)_ = .004, *p* = .949	φ = .006
	M	(SD)	M	(SD)	M	(SD)		
diabetes-related distress^d^	8.6	(5.1)	10.4	(5.0)	6.8	(4.7)	*t*_(112)_ = 4.033, *p* <.001	d = .756
personality functioning^e^	18.6	(11.1)	23.6	(11.4)	13.9	(8.4)	*t*_(100,857)_ = 5.136, *p* <.001	d = .967
epistemic trust^f^	24.6	(5.2)	24.8	(5.4)	24.4	(5.1)	*t*_(111)_ = .409, *p* = .683	d = .077
epistemic mistrust^f^	11.9	(4.3)	13.4	(3.8)	10.6	(4.4)	*t*_(111)_ = 3.677, *p* <.001	d = .692
epistemic credulity^f^	13.5	(5.7)	16.0	(5.6)	11.1	(4.6)	*t*_(111)_ = 5.000, *p* <.001	d = .941

^a^Different types of child maltreatment were assessed with the self-report questionnaire Childhood Trauma Questionnaire (CTQ). Multiple answers were possible. ^b^Psychological burden was assessed with the German version of the Patient-Health-Questionnaire (PHQ-D). ^c^Given that the expected cell frequencies were less than five, the χ^2^-test could not be interpreted. ^d^Diabetes-related distress was assessed with the 5-item short form of the Problem Areas in Diabetes Scale (PAID-5). Range: 0 to 20. ^e^Personality functioning was assessed with the Operationalized Psychodynamic Diagnosis – Structure Questionnaire Short Form (OPD-SQS). Range: 0 to 48. ^f^Epistemic trust, epistemic mistrust, and epistemic credulity were assessed with the German 12-item version of the Epistemic Trust, Mistrust and Credulity Questionnaire (ETMCQ). Range trust: 5 to 35, range mistrust: 3 to 21, range credulity: 4 to 28. n.a.= not available.

Supplementary subgroup analyses compared patients with type 1 diabetes and child maltreatment (n=24) to patients with type 1 diabetes and no child maltreatment (n=31) as well as patients with type 2 diabetes and child maltreatment (n=32) to patients with type 2 diabetes and no child maltreatment (n=27). Patients with type 1 diabetes and child maltreatment compared to those without reported significantly higher diabetes-related distress (*t*_(54)_=3.977, *p* <.001, d=1.081) with a large effect size as well as higher frequencies of major depressive syndrome (χ^2^_(1)_=4.441, *p*=.035, φ=.284) and somatoform syndrome (χ^2^_(1)_=6.139, *p*=.013, φ=.334), with small to medium effect sizes. See also **Supplementary Table 1**. In patients with type 2 diabetes, those with child maltreatment compared to those without also showed significantly higher frequencies of major depressive syndrome (χ^2^_(1)_=6.331, *p*=.012, φ=.328) and other anxiety syndromes (χ^2^_(1)_=5.138, *p* = .023, φ=.295) with medium and small effect sizes respectively. However, there were no differences regarding diabetes-related distress (*t*_(57)_=1.931, *p* = .058, d=.505), with a large effect size. See also **Supplementary Table 2**.

### 3.3 Differences in personality functioning and epistemic trust, mistrust, and credulity according to a history of child maltreatment

In the total sample, results show that impairments in personality functioning are more severe in those with child maltreatment compared to those without (*t*_(100,857)_=5.136, *p* <.001, d=.967), with a large effect size. Further, epistemic mistrust and credulity as measured by the ETMCQ-12 were significantly higher in patients with diabetes and child maltreatment compared to those without (mistrust: *t*_(111)_=3.677, *p* <.001, d=.692; credulity: *t*_(111)_=5.000, *p* <.001, d=.941), with medium and large effect sizes respectively. We found no differences regarding epistemic trust (*t*_(111)_=.409, *p=*.683, d=.077), with the effect size falling below the threshold for small effects. See also **Table 2**.

Subgroup analyses differentiated patients with type 1 and type 2 diabetes, and examined differences in personality functioning, epistemic trust, mistrust, and credulity in those with and without child maltreatment. In patients with type 1 diabetes, differences between those with and without child maltreatment emerged regarding personality functioning (*t*_(53)_=3.336, *p=*.002, d=.907) and epistemic credulity (*t*_(53)_=3.164, *p=*.003, d=.860), with large effect sizes. However, no differences regarding epistemic trust and mistrust could be observed (trust: *t*_(53)_=.316, *p=*.753, d=.086; mistrust: *t*_(53)_=1.708, *p=*.093, d=.464), with the effect size falling below the threshold for small effects regarding trust, and a small effect size for mistrust. See also **Supplementary Table 1**. In patients with type 2 diabetes, those with child maltreatment compared to those without showed higher impairments in personality functioning (*t*_(55,703)_=4.147, *p* <.001, d=1.054), epistemic mistrust (*t*_(41,398)_=3.191, *p=*.003, d=.868), and epistemic credulity (*t*_(56)_=3.761, *p* <.001, d=.990), with large effect sizes. No differences were observed regarding epistemic trust (*t*_(56)_=.318, *p=*.751, d=.084), however, the effect size falls below the threshold for small effects. See also **Supplementary Table 2**.

### 3.4 Regression and mediation analyses

To better understand the association between heightened diabetes-related distress and more frequent psychological burden in patients with diabetes and child maltreatment compared to those without, we conducted regression and mediation analyses. Correlation analyses for all variables can be found in **Table 3**.

**Table 3:** Correlations with bootstrapped confidence intervals.

	CTQ sexual abuse	CTQ emotional abuse	CTQ physical abuse	CTQ emotional neglect	CTQ physical neglect	CTQ sum score	OPD-SQS	ETMCQ trust	ETMCQ mistrust	ETMCQ credulity	PHQ-9	PHQ-15
CTQ sexual abuse^a^	–											
CTQ emotional abuse^a^	.342^**^	–										
	[.094,.540]											
CTQ physical abuse^a^	.303^**^	.542^**^	–									
	[-.055,.643]	[.301,.731]										
CTQ emotional neglect^a^	.327^**^	.666^**^	.408^**^	–								
	[.122,.487]	[.531,.782]	[.224,.575]									
CTQ physical neglect^a^	.328^**^	.475^**^	.481^**^	.665^**^	–							
	[-.061,.593]	[.272,.653]	[.186,.699]	[.541,.769]								
CTQ sum score^a^	.568^**^	.848^**^	.742^**^	.826^**^	.751^**^	–						
	[.219,.747]	[.788,.897]	[.573,.847]	[.757,.884]	[.591,.855]							
OPD-SQS^b^	.233^*^	.527^**^	.255^*^	.463^**^	.262^*^	.484^**^	–					
	[.017,.435]	[.361,.673]	[.109,.420]	[.262,.635]	[.072,.447]	[.330,.636]						
ETMCQ trust^c^	-.123	-.003	.058	-.093	.092	-.019	-.166	–				
	[-.340,.110]	[-.225,.194]	[-.185,.281]	[-.285,.092]	[-.119,.280]	[-.237,.179]	[-.423,.100]					
ETMCQ mistrust^c^	.095	.285^**^	.104	.163	.136	.219^*^	.516^**^	.003	–			
	[-.039,.222]	[.067,.487]	[-.108,.346]	[-.051,.364]	[-.029,.296]	[.034,.402]	[.337,.671]	[-.258,.259]				
ETMCQ credulity^c^	.189	.429^**^	.289^**^	.344^**^	.318^**^	.428^**^	.555^**^	-.040	.562^**^	–		
	[.038,.319]	[.224,.600]	[.132,.439]	[.124,.537]	[.146,.474]	[.254,.588]	[.370,.708]	[-.290,.209]	[.374,.712]			
PHQ-9^d^	.126	.349^**^	.274^*^	.400^**^	.195	.375^**^	.679^**^	-.084	.359^**^	.395^**^	–	
	[-.048,.289]	[.157,.545]	[.042,.499]	[.223,.579]	[-.004,.400]	[.194,.572]	[.538,.788]	[-.346,.191]	[.150,.538]	[.198,.553]		
PHQ-15^e^	.150	.342^**^	.220^*^	.304^**^	.090	.315^**^	.553^**^	-.072	.287^**^	.281^**^	.760^**^	–
	[-.025,.321]	[.163,.503]	[.028,.412]	[.115,.482]	[-.109,.301]	[.137,.499]	[.363,.699]	[-.271,.123]	[.073,.475]	[.093,.454]	[.645,.846]	
PAID-5^f^	.089	.304^**^	.224^*^	.266^*^	.165	.294^**^	.498^**^	-.019	.351^**^	.369^**^	.688^**^	.668^**^
	[-.121,.302]	[.135,.475]	[.019,.426]	[.084,.442]	[-.068,.381]	[.096,.490]	[.327,.650]	[-.247,.216]	[.158,.519]	[.213,.511]	[.575,.787]	[.527,.782]

^a^Different types of child maltreatment were assessed with the self-report questionnaire Childhood Trauma Questionnaire (CTQ). Multiple answers were possible. ^b^Personality functioning was assessed with the Operationalized Psychodynamic Diagnosis – Structure Questionnaire Short Form (OPD-SQS). Range: 0 to 48. ^c^Epistemic trust, epistemic mistrust, and epistemic credulity were assessed with the German 12-item version of the Epistemic Trust, Mistrust and Credulity Questionnaire (ETMCQ). Range trust: 5 to 35, range mistrust: 3 to 21, range credulity: 4 to 28. ^d^Depressive symptoms and their severity level were assessed with the PHQ-9 as part of the PHQ-D. Range: 0 to 27. ^e^Somatic symptoms and their severity level were assessed with the PHQ-15 as part of the PHQ-D. Range: 0 to 30. ^f^Diabetes-related distress was assessed with the 5-item short form of the Problem Areas in Diabetes Scale (PAID-5). Range: 0 to 20. *p*-values: *p ≤.05. **p ≤.01. ***p ≤.001. Point-biserial correlations were computed with 95% bootstrapped confidence intervals based on 5,000 bootstrap samples. Coefficients >.1 indicate weak correlation, >.3 indicate moderate correlation, and >.5 indicate strong correlation.

**Figure 1:**
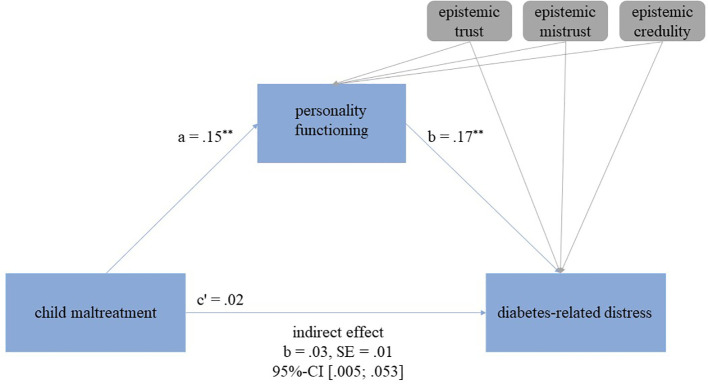
Mediation model examining the association between child maltreatment and diabetes-related distress as well as personality functioning as a mediator and epistemic trust, mistrust, and credulity as covariates. The indirect (mediating) effect is present, if the confidence intervals (CI) exclude zero. *p*-values: ^*^ *p* ≤ 0.05, ^**^ *p* ≤ 0.01, ^***^ *p* ≤ 0.001.

In the regression analysis, a significant direct association between child maltreatment and diabetes-related distress emerged, showing that higher scores of child maltreatment were associated with higher diabetes-related distress (adjusted R^2^=.094; b=.10, 95%-CI [.044,.153], *p* <.001). In the mediation analysis, the direct was not significant anymore (b=.2, 95%-CI [-.032,.081], *p* =.387). The overall explained variance in the model increased from 9.4% in the regression analysis to 24.3% in the mediation model. An indirect effect of personality functioning between child maltreatment and diabetes-related distress was found (b=.03, 95%-CI [.005,.053]), indicating partial mediation. Epistemic trust, mistrust, and credulity were significantly associated with personality functioning (trust: b=-.33, 95%-CI [-.619, -.032], *p*=.030; mistrust: b=.75, 95%-CI [.306, 1.203], *p*=.001; credulity: b=.61, 95%-CI [.244,.971], *p*=.001). However, only mistrust was associated with diabetes-related distress (mistrust: b=.37, 95%-CI [.112,.620], *p*=.005). There was no association between trust (b=-.07, 95%-CI [-.235,.097], *p*=.410) and credulity (b=.12, 95%-CI [-.089,.323], *p*=.262), and diabetes-related distress. See also **Figure 1**.

**Figure 2:**
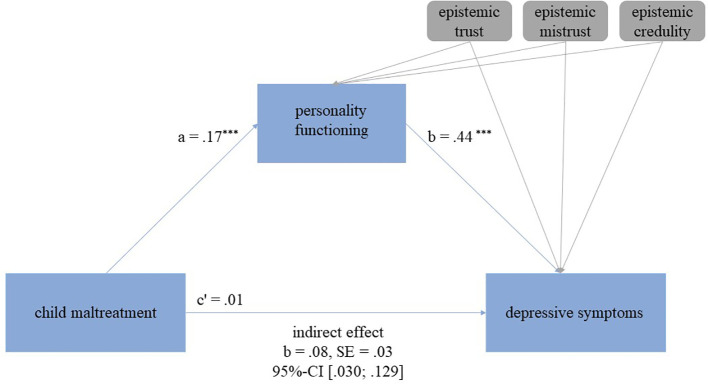
Mediation model examining the association between child maltreatment and depressive symptoms as well as personality functioning as a mediator and epistemic trust, mistrust, and credulity as covariates. The indirect (mediating) effect is present, if the confidence intervals (CI) exclude zero. *p*-values: ^*^ *p* ≤ 0.05, ^**^ *p* ≤ 0.01, ^***^ *p* ≤ 0.001.

In the regression analysis, we observed a significant direct effect, showing that higher scores of child maltreatment were associated with higher depressive symptoms (adjusted R^2^=.132; b=.15, 95%-CI [.080,.228], *p* <.001). In the mediation analysis, the direct effect was no longer significant (b=.01, 95%-CI [-.052,.077], *p*=.695). The overall explained variance in the model increased from 13.2% in the regression model to 52.7% in the mediation model. An indirect effect of personality functioning emerged (b=.08, 95%-CI [.030,.129]), indicating full mediation. Epistemic trust, mistrust, and credulity were significantly associated with personality functioning (trust: b=-.30, 95%-CI [-.584, -.013], *p*=.041; mistrust: b=.87, 95%-CI [.426, 1.316], *p≤*.001; credulity: b=.53, 95%-CI [.165,.893], *p*=.005), but not with depressive symptoms (trust: b=.03, 95%-CI [-.150,.209], *p*=.743; mistrust: b=-.00, 95%-CI [-.298,.289], *p*=.977; credulity: b=.01, 95%-CI [-.241,.225], *p*=.946). See also **Figure 2**.

**Figure 3:**
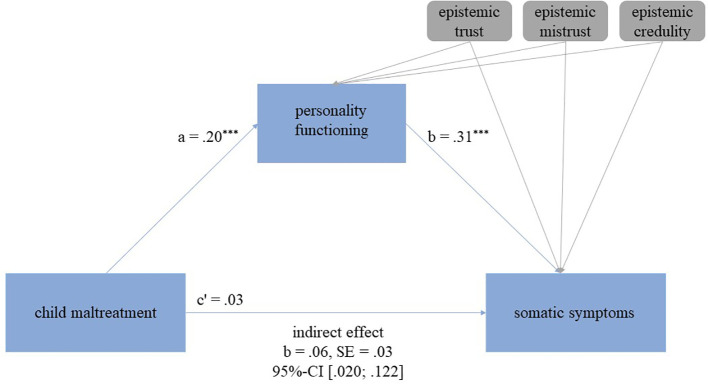
Mediation model examining the association between child maltreatment and somatic symptoms as well as personality functioning as a mediator and epistemic trust, mistrust, and credulity as covariates. The indirect (mediating) effect is present, if the confidence intervals (CI) exclude zero. *p*-values: ^*^ *p* ≤ 0.05, ^**^ *p* ≤ 0.01, ^***^ *p* ≤ 0.001.

In the regression analysis, a significant direct association was also found between child maltreatment and somatic symptoms, showing that higher scores of child maltreatment were associated with higher somatic symptoms (adjusted R^2^=.090; b=.11, 95%-CI [.041,.187], *p*=.003). In the mediation analysis however, the direct effect was no longer significant (b=.03, 95%-CI [-.052,.106], *p*=.494). The overall explained variance in the model increased from 9.0% in the regression model to 31.5% in the mediation model. An indirect effect of personality functioning emerged (b=.06, 95%-CI [.020,.122]), indicating full mediation. While epistemic trust was not significantly associated with personality functioning (b=-.29, 95%-CI [-.595,.020], *p*=.067), mistrust, and credulity were (mistrust: b=.76, 95%-CI [.286, 1.239], *p*=.002; credulity: b=.46, 95%-CI [.084,.842], *p*=.017). Neither epistemic trust, mistrust, nor credulity were associated with somatic symptoms (trust: b=.02, 95%-CI [-.188,.222], *p*=.866; mistrust: b=.04, 95%-CI [-.288,.371], *p*=.804; credulity: b=-.07, 95%-CI [-.329,.184], *p*=.575). See also **Figure 3**.

## 4 Discussion

This article shows that child maltreatment is a frequently reported phenomenon in a clinical sample of patients with type 1 and type 2 diabetes. Comparing patients with diabetes with and without a history of child maltreatment shows that those with child maltreatment report higher diabetes-related distress and psychological burden as well as higher impairments in personality functioning, higher epistemic mistrust, and epistemic credulity.

In a clinical sample of patients with type 1 and type 2 diabetes, 32.8% reported elevated diabetes-related distress and 53.8% met the categorical self-report threshold for at least one type of psychological burden, with 26.9% meeting the threshold for two or more types of psychological burden. While data on the prevalence of diabetes-related distress especially in clinical samples is limited, our findings add evidence to the higher frequency in clinical samples compared to population-based samples (18, 19). However, our frequencies for psychological burden exceed those from a comparable German clinical sample of patients with type 1 and type 2 diabetes, showing that around 39% screen positively for a mental disorder (56). Contrary to other diabetes populations, we had higher percentages of males than females. While female patients with diabetes tend to suffer from psychosocial aspects more frequently than men (57), our findings indicate that especially male patients have difficulties regulating their blood sugar levels and show comorbid diabetes-related distress and psychological burden, and hence, seek support from a specialized diabetes clinic, which we recruited the sample of the present investigation from. Moreover, the frequency of at least one type of self-reported child maltreatment with at least moderate severity was 49.1%, and 37.0% reported at least one type of severe child maltreatment. These frequencies are remarkably higher compared to the frequencies found in patients with diabetes from population-based samples (22). Patients with type 2 diabetes reported significantly more often physical abuse compared to patients with type 1 diabetes, even though effect sizes were small. However, this corresponds to findings that underline in particular a common occurrence of child maltreatment in type 2 diabetes (58, 59).

First, we investigated whether patients with diabetes and a history of child maltreatment compared to patients with diabetes without child maltreatment show higher diabetes-related distress as well as more frequent psychological burden. To our knowledge, the direct association between child maltreatment and diabetes-related distress has not been studied so far, and regarding psychological burden, research investigating associations between child maltreatment in samples of patients with diabetes is still limited. Studies employing e.g., population-based data have abundantly emphasized the negative impact of child maltreatment on e.g., symptoms of depression and anxiety, or somatic symptoms (29, 60). Accordingly, we show that in a clinical sample of patients with type 1 and type 2 diabetes, a history of child maltreatment is significantly associated with high psychological burden in general, and depressive and somatic symptoms in particular, with small to medium effect sizes.

We further examined if patients with diabetes and a history of child maltreatment show higher impairments in personality functioning and epistemic trust, mistrust, and credulity compared to patients with diabetes without child maltreatment. We consistently found higher impairments in personality functioning in those with a history of child maltreatment compared to those without with medium to large effect sizes. This is in line with previous studies showing the negative impact of child maltreatment on personality functioning not only in the general population, but also in patients with diabetes (22). Regarding the epistemic stance, we only found higher epistemic mistrust and credulity in those with child maltreatment compared to those without, but no differences regarding epistemic trust. Similar results were found in the association between ACEs and symptoms of posttraumatic stress disorder (PTSD), with impaired personality functioning, epistemic mistrust and credulity – but not epistemic trust – being associated with PTSD (35). This supports the conception that epistemic trust is not simply the inverse of epistemic mistrust and credulity, but rather a distinct psychological capacity, which might not necessarily be significantly reduced by e.g., child maltreatment. If the ability for epistemic trust can generally be preserved after child maltreatment, this may also pose a starting point for preventative measures. However, more research is needed to better understand how child maltreatment affects epistemic trust, mistrust, and credulity. In patients with diabetes, the epistemic stance is only scarcely researched. However, the relevance of trust in general was outlined in e.g., a multicentre study, where higher trust in physicians was associated with reduced symptoms of depression and anxiety in patients with diabetes, and further, the predictive value of trust in physicians for adherence was identified (61, 62). Hence, we provide preliminary evidence that promoting epistemic trust in patients with diabetes might pose a useful new element in diabetes treatment.

Lastly, we examined if the association between child maltreatment and diabetes-related distress, and between child maltreatment and psychological burden is mediated by personality functioning, including epistemic trust, mistrust, and credulity as covariates. We found an indirect (mediating) effect of personality functioning in the association between child maltreatment and diabetes-related distress (partial mediation), and between child maltreatment and depressive symptoms as well as somatoform symptoms (full mediation). These findings relate to studies conducted in the general population, showing a mediating effect of personality functioning in the association between child maltreatment and e.g., symptoms of depression and anxiety, and somatic symptom burden (28, 29). To our knowledge, we are the first to investigate a mediating effect of personality functioning in the association between child maltreatment and diabetes-related distress, and between child maltreatment and psychological burden, respectively in a sample of patients with diabetes. Our findings indicate that the high diabetes-related distress and psychological burden in a clinical sample of patients with diabetes is partly conveyed by impaired personality functioning. These findings strengthen the perspective that impairments in personality functioning may also reflect the reduced abilities to meet the demands of coping with the diabetes disease, making these individuals more vulnerable for associated increased diabetes-related distress and psychological burden. However, personality functioning has shown to be improved by psychotherapy (63). Following this line of thinking, by targeting impaired personality functioning in patients with diabetes, overall psychological abilities to cope with diabetes and its demands might be strengthened. Thereby, diabetes-related distress and psychological burden might be reduced, which is of great clinical importance in the high-risk group of patients with diabetes. Despite the preliminary findings presented in this study, this notion remains to be investigated.

### 4.1 Strengths and limitations

The present study is based on a clinical sample of individuals with a medically diagnosed type 1 and type 2 diabetes, offering insights regarding diabetes-related distress and psychological burden in this high-risk patient group. Next to providing frequencies of at least moderate severity of self-reported child maltreatment, diabetes-related distress as well as psychological burden, we also present impairments in personality functioning as a relevant mechanism, significantly expanding existing knowledge on this topic.

However, this study has certain limitations. First, the cross-sectional data does not allow for causal interpretations of the findings. Mediation analyses are prone to several methodological and conceptual challenges, for example misspecification of the model or unmeasured confounding (64). Due to the established temporal nature of child maltreatment as well as the strong empirical evidence demonstrating the developmental impact of early adversity on mental health, sufficient plausibility for the assumed causal relationships in the mediation model is given. Second, the sample sizes for the subgroup analyses comparing patients with type 1 diabetes with and without child maltreatment and patients with type 2 diabetes with and without child maltreatment are relatively small and therefore, must be interpreted with caution. Further, the use of self-report instruments, especially regarding self-reported child maltreatment, is susceptible to bias. However, studies indicate that subjective reports of child maltreatment are associated with psychopathology, independently from objective reports (65). As one important objective of the present study was to investigate the association between child maltreatment and psychological burden, self-report measures seem to be suitable. While the CTQ that was used in the present study to retrospectively assess self-reported child maltreatment is a reliable and valid screening instrument, the cut-offs used to determine if a type of child maltreatment is present or not (at least moderate severity) are not validated. However, using the cut-off of at least moderate severity is common in German prevalence studies of child maltreatment (43, 44). Future research could consider other confounding factors regarding diabetes-related distress and psychological burden, both sociodemographic (e.g. household income, migration background, education), and diabetes-related (diabetes duration, comorbid secondary diseases, use of antidepressants), use longitudinal data to support our preliminary findings as well as conduct subgroup analyses of patients with type 1 and type 2 diabetes in larger samples.

### 4.2 Conclusion

In a clinical sample of patients with type 1 and type 2 diabetes, the frequency of diabetes-related distress and psychological burden, especially in terms of depressive and somatoform symptoms, is high. This is aggravated by a history of child maltreatment. Further, patients with diabetes and a history of child maltreatment compared to those without a history of child maltreatment also show higher impairments in personality functioning and increased epistemic mistrust and credulity. The association between child maltreatment and diabetes-related distress, as well as between child maltreatment and depressive symptoms, and somatic symptoms respectively is mediated by personality functioning. Hence, impairments in personality functioning in patients with diabetes might point to a reduced emotional and interpersonal ability in dealing with the disease, providing a novel underlying pathway between child maltreatment and increased diabetes-related distress and psychological burden. Screening for impairments in personality functioning in routine diabetes care could help initiating targeted interventions such as psychodynamic psychotherapy or mentalization-based psychotherapy, which focus on strengthening aspects of personality functioning, and provide a new treatment avenue for highly burdened patients with diabetes.

## Data Availability

The raw data supporting the conclusions of this article will be made available by the authors, without undue reservation.
