# Supplementary material for: Diabetes-related distress and psychological burden in patients with type 1 and type 2 diabetes – the role of child maltreatment, personality functioning, and epistemic trust: findings from a German clinical inpatient sample
Source: Front Psychiatry. 2025 Jul 17;16:1608601. doi: 10.3389/fpsyt.2025.1608601 (PMC12310633; doi:10.3389/fpsyt.2025.1608601)
Supplement: Supplementary file 1 [file Table1.docx]

**Supplemental Table 1. Differences in diabetes-related distress and psychological burden as well as personality functioning, and epistemic trust according to a history of child maltreatment in patients with type 1 diabetes**

|  | **total sample**  **(n = 55)** | | **child maltreatment^a^**  **(n = 24)** | | **no child maltreatment**  **(n = 31)** | | **test statistics** | **effect size** |
| --- | --- | --- | --- | --- | --- | --- | --- | --- |
| **psychological burden**^b^ | **N** | **(%)** | **N** | **(%)** | **N** | **(%)** |  |  |
| major depressive syndrome | 17 | (30.9) | 11 | (45.8) | 6 | (19.4) | **χ^2^_(1)_ = 4.441, *p* = .035** | **φ = .284** |
| somatoform syndrome | 12 | (21.8) | 9 | (37.5) | 3 | (9.7) | **χ^2^_(1)_ = 6.139, *p* = .013** | **φ = .334** |
| panic syndrome | 5 | (9.1) | 4 | (16.7) | 1 | (3.2) | n.a.^c^ |  |
| other anxiety syndromes | 5 | (9.1) | 4 | (16.7) | 1 | (3.2) | n.a.^c^ |  |
| binge-eating disorder | 8 | (14.8) | 5 | (20.8) | 3 | (10.0) | n.a.^c^ |  |
| alcohol syndrome | 8 | (14.5) | 4 | (16.7) | 4 | (12.9) | n.a.^c^ |  |
|  | **M** | **(SD)** | **M** | **(SD)** | **M** | **(SD)** |  |  |
| diabetes-related distress^d^ | 8.4 | (5.0) | 11.0 | (5.1) | 6.3 | (3.8) | ***t*_(53)_ = 3.977, *p* < .001** | **d = 1.081** |
| personality functioning^e^ | 19.1 | (10.8) | 24.2 | (11.5) | 15.2 | (8.4) | ***t*_(53)_ = 3.336*,***  ***p* = .002** | **d = .907** |
| epistemic trust^f^ | 24.8 | (5.1) | 25.1 | (5.4) | 24.6 | (4.9) | *t*_(53)_ = .316,  *p* = .753 | d = .086 |
| epistemic mistrust^f^ | 11.7 | (3.9) | 12.7 | (4.3) | 10.9 | (3.3) | *t*_(53)_ = 1.708, *p* = .093 | d = .464 |
| epistemic credulity^f^ | 13.1 | (5.3) | 15.3 | (5.1) | 11.3 | (4.3) | ***t*_(53)_ = 3.164, *p* = .003** | **d = .860** |

*Notes.* ^a^ Different types of child maltreatment were assessed with the self-report questionnaire Childhood Trauma Questionnaire (CTQ). Multiple answers were possible. ^b^ Psychological burden was assessed with the German version of the Patient-Health-Questionnaire (PHQ-D). ^c^ Given that the expected cell frequencies were less than five, the χ^2^-test could not be interpreted. ^d^Diabetes-related distress was assessed with the 5-item short form of the Problem Areas in Diabetes Scale (PAID-5). Range: 0 to 20.  ^e^ Personality functioning was assessed with the Operationalized Psychodynamic Diagnosis – Structure Questionnaire Short Form (OPD-SQS). Range: 0 to 48. ^f^Epistemic trust, epistemic mistrust and epistemic credulity were assessed with the German 12-item version of the Epistemic Trust, Mistrust and Credulity Questionnaire (ETMCQ). Range trust: 5 to 35, range mistrust: 3 to 21, range credulity: 4 to 28.
