# Supplementary material for: Diabetes-related distress and psychological burden in patients with type 1 and type 2 diabetes – the role of child maltreatment, personality functioning, and epistemic trust: findings from a German clinical inpatient sample
Source: Front Psychiatry. 2025 Jul 17;16:1608601. doi: 10.3389/fpsyt.2025.1608601 (PMC12310633; doi:10.3389/fpsyt.2025.1608601)
Supplement: Supplementary file 2 [file Table2.docx]

**Supplemental Table 2. Differences in diabetes-related distress and psychological burden as well as personality functioning, and epistemic trust according to a history of child maltreatment in patients with type 2 diabetes**

|  | **total sample**  **(n = 59)** | | **child maltreatment^a^**  **(n = 32)** | | **no child maltreatment**  **(n = 27)** | | **test statistics** | **effect size** |
| --- | --- | --- | --- | --- | --- | --- | --- | --- |
| **psychological burden**^b^ | **N** | **(%)** | **N** | **(%)** | **N** | **(%)** |  |  |
| major depressive syndrome | 21 | (35.6) | 16 | (50.0) | 5 | (18.5) | **χ^2^_(1)_ = 6.331, *p* = .012** | **φ = .328** |
| somatoform syndrome | 19 | (32.2) | 13 | (40.6) | 6 | (22.2) | χ^2^_(1)_ = 2.272, *p* = .132 | φ = .196 |
| panic syndrome | 7 | (11.9) | 6 | (18.8) | 1 | (3.7) | n.a.^c^ |  |
| other anxiety syndromes | 12 | (20.3) | 10 | (31.3) | 2 | (7.4) | **χ^2^_(1)_ = 5.138, *p* = .023** | **φ = .295** |
| bulimia nervosa | 2 | (3.4) | 2 | (6.3) | 0 | (0.0) | n.a.^c^ |  |
| binge-eating disorder | 7 | (13.5) | 4 | (13.8) | 3 | (13.0) | n.a.^c^ |  |
| alcohol syndrome | 4 | (6.8) | 2 | (6.3) | 2 | (7.4) | n.a.^c^ |  |
|  | **M** | **(SD)** | **M** | **(SD)** | **M** | **(SD)** |  |  |
| diabetes-related distress^d^ | 8.7 | (5.4) | 9.9 | (5.0) | 7.3 | (5.5) | *t*_(57)_ = 1.931, *p* = .058 | d = .505 |
| personality functioning^e^ | 18.2 | (11.4) | 23.1 | (11.5) | 12.4 | (8.3) | ***t*_(55,703)_ = 4.147*,***  ***p* < .001** | **d = 1.054** |
| epistemic trust^f^ | 24.4 | (5.4) | 24.6 | (5.5) | 24.2 | (5.5) | *t*_(56)_ = .318,  *p* = .751 | d = .084 |
| epistemic mistrust^f^ | 12.2 | (4.7) | 14.0 | (3.2) | 10.2 | (5.4) | ***t*_(41,398)_ = 3.191, *p* = .003** | **d = .868** |
| epistemic credulity^f^ | 13.9 | (6.2) | 16.5 | (6.1) | 10.9 | (5.0) | ***t*_(56)_ = 3.761,**  ***p* < .001** | **d = .990** |

*Notes.* ^a^ Different types of child maltreatment were assessed with the self-report questionnaire Childhood Trauma Questionnaire (CTQ). Multiple answers were possible. ^b^ Psychological burden was assessed with the German version of the Patient-Health-Questionnaire (PHQ-D). ^c^ Given that the expected cell frequencies were less than five, the χ^2^-test could not be interpreted. ^d^Diabetes-related distress was assessed with the 5-item short form of the Problem Areas in Diabetes Scale (PAID-5). Range: 0 to 20.  ^e^ Personality functioning was assessed with the Operationalized Psychodynamic Diagnosis – Structure Questionnaire Short Form (OPD-SQS). Range: 0 to 48. ^f^Epistemic trust, epistemic mistrust and epistemic credulity were assessed with the German 12-item version of the Epistemic Trust, Mistrust and Credulity Questionnaire (ETMCQ). Range trust: 5 to 35, range mistrust: 3 to 21, range credulity: 4 to 28.
